# Periprosthetic humeral fractures after reverse shoulder arthroplasty. Case report

**DOI:** 10.1590/s1679-45082017rc4037

**Published:** 2017-09-04

**Authors:** Andre Wajnsztejn, Noel Oizerovici Foni, Dan Oizerovici, Robinson Esteves Santos Pires, Benno Ejnisman

**Affiliations:** 1 Hospital Israelita Albert Einstein, São Paulo, SP, Brazil.; 2 Universidade Federal de Minas Gerais, Belo Horizonte, MG, Brazil.; 3 Escola Paulista de Medicina, Universidade Federal de São Paulo, São Paulo, SP, Brazil.

**Keywords:** Humeral fractures, Arthroplasty, Case reports, Fraturas do úmero, Artroplastia, Relatos de casos

## Abstract

Periprosthetic fractures is a severe complication after joint replacement. The rapidly increase of reverse shoulder arthroplasty surgeries, periprosthetic humeral fractures, which are described as rare, may increase in the near future. We report the case of displaced humeral fracture bellow the stem of reverse shoulder prosthesis. The patient was an 85-year-old woman who had a total shoulder replacement 6 years previously. The surgical solution consisted of plate osteossynthesis and cerclage. This report describes an unprecedented case in Brazilian literature; and gives an overview of the existing literature including this injury classification.

## RESUMO

As fraturas periprotéticas do ombro representam uma grave complicação após a substituição da articulação. Devido ao rápido aumento do número de cirurgias de artroplastia reversa no ombro, as fraturas periprótese do úmero, descritas como raras, podem aumentar no futuro próximo. Descrevemos um caso de fratura desviada do úmero abaixo do componente umeral de uma artroplastia reversa. A paciente era uma mulher de 85 anos de idade, que tinha uma artroplastia reversa do ombro há 6 anos. O tratamento cirúrgico consistiu em osteossíntese com placa, parafusos e cerclagem. Os objetivos do presente trabalho foram reportar um caso inédito na literatura brasileira, e revisar a literatura existente e as classificações da lesão.

## INTRODUCTION

The incidence of periprosthetic fractures after reverse shoulder arthroplasty (RSA) is around 2%.^( 1 )^ This is a rare fracture and often occurs in osteoporotic patients, older persons, and among those who have a variety of comorbidities.

Biomechanical characteristics of RSA increase the number of diseases in which this procedure can be used, such as irreversible rotator cuff injuries, shoulder arthritis and proximal humerus extremity fractures. For this reason, the number of RSA surgeries have increased significantly.^( 2 )^

Technical difficult for treatment of perisprosthetic fractures after RSA surgery, its features, patient’s comorbidities, the small number of cases and scarcity of studies in national literature justifies this case report.

## CASE REPORT

**Figure 1:**
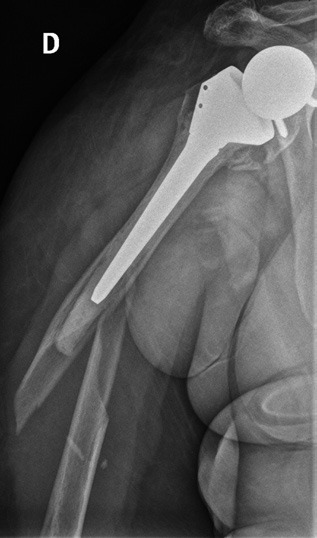
Front radiography showing reverse shoulder periarthroplasty fracture

This was an 85-year-old woman, right-handed, insulin-dependent, hypertensive, obese with body mass index of 42, who had fell over her right upper limb ( Figure 1 ). She did not have any neurovascular changes after the fall.

Patient had a cemented RSA performed 6 years before the fracture because of massive rotator cuff injury. Before the fracture, she had an active arch elevation motion of 60°, abduction of 50°, lateral rotation of 40° and medial of 50°.

We did not observed loosening of RSA components, according to criteria defined by Sanchez-Sotelo et al.^( 3 )^

The non-surgical management of the injury was discussed with patient. However, because of her clinical conditions (obesity and diabetes) e better control of pain, we decided for the surgery.

**Figure 2:**
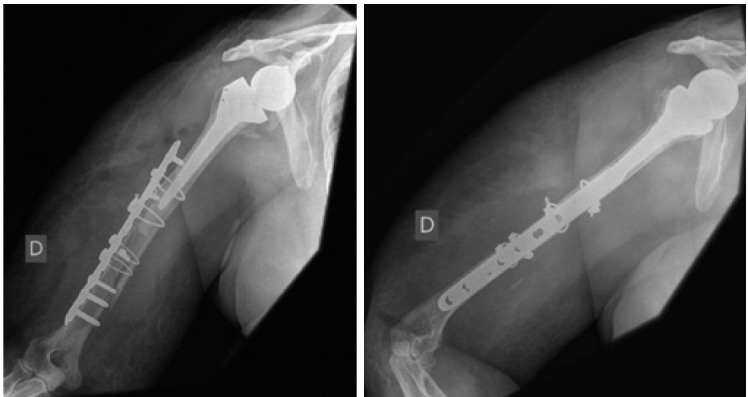
Front and profile radiography showing osteosynthesis with plates, screws and cables immediate after surgery

In surgery, we used lateral approach due to possibility of proximal extension access, and visualization and protection of radial nerve all the way along its course. A 3.5mm locking plate (DePuy, Synthes^®^) associated with cerclage and osteosynthesis with loops were used ( Figure 2 ).

**Figure 3:**
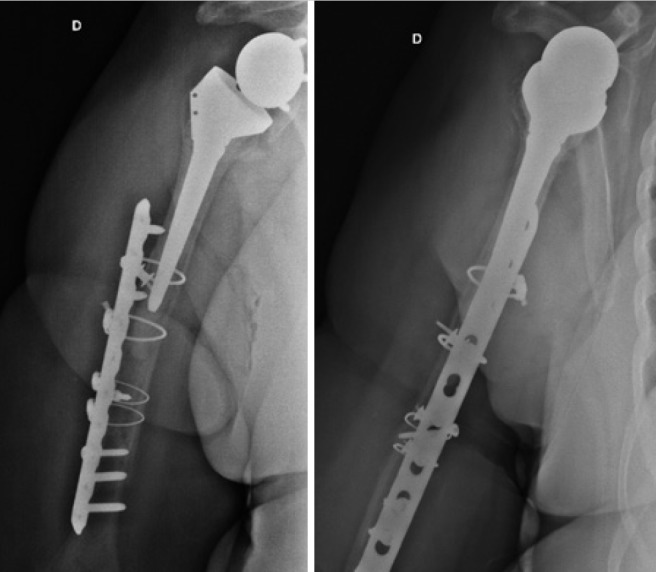
Front and profile radiography, showing fracture healing

Patient used a simple sling for 2 weeks, and an immobilized elbow to 90° degree, whereas passive movements of the limb were done. In the third week, the patient begun active movements, and 3 months after surgery, she evolved with consolidation of the fracture and the limb regained the same function level it had before the surgery ( Figure 3 ).

In an one year follow-up after the procedure, we evaluated patient function based on Disabilities of the Arm, Shoulder and Hand (DASH) index that was translated into Brazilian Portuguese,^( 4 )^ her score was 78, being 0 the best score and 100 is worse.

Patient’s active arch motion reduced for degree of elevation of 45°, abduction of 40°, lateral rotation of 40° and medial of 30°.

## DISCUSSION

Recently, the RSA surgery has become popular^( 2 )^ and, consequently, complications related to the procedure have also increased.

Post-operative trauma fractures in shoulder prosthesis, including partial and total arthroplasties are rare injuries. They occur in around 0.6 and 3% of cases or shoulder prosthesis.^( 4 )^

The RSA is often indicated for irreversible rotator cuff injuries, complex proximal humerus extremity fractures and arthritis. In general, patient that requires the RSA is older and they have several comorbidities, such as diabetes, obesity, hypertension, cardiovascular and pulmonary diseases. No studies exist on prevalence of periprothesis fractures in RSA. Most of studies approach complications of all shoulder arthroplasties, including partial and total prosthesis. In a series described by García-Fernandéz et al.,^( 1 )^ including 203 RSA, only 4 patients had periprothesis fractures in the post-operative period. Of these, 3 were treated surgically using plates and screws associated with cerclage, and 1 was treated conservatively. In a series of Bacle et al.,^( 5 )^ 191 RSA had mean follow-up of 40 months, no patient had post-operative fracture.

There is a trend of surgical treatment of obese patients for their low tolerance to immobilization methods. Clinical conditions of our patient led to increase of surgical time, blood loss and hospital costs.^( 6 )^ Obese patients often have a longer post-operative period than non-obese patients.

Surgical treatment can be done using two techniques: RSA review with longer humeral stem or osteosynthesis. In general, the RSA review is indicated when prosthesis has signs of looseness.^( 7 )^

Osteosynthesis can be done using posterior or lateral approach. Both accesses enable visualization and protection of radial nerve, but lateral approach enable a possible extension of proximal access, in case that need longer fixation. Identification of nerve is need for its protection and to perform cerclage.

Fixation can be done with plates and screws, plates and cerclage, and plates and screws associated with cerclage. Locked screws promote rotational control, and cerclage increases the stability of all construction – that is reason that this technique was chosen.

A number of classifications are used for periprosthetic fractures of the humerus. Wright et al.,^( 8 )^ divide fractures into 3 types (A, B and C) based in nine cases: type A if long trait proximal to stem with length of at least one third of the stem size, type B if the short trait proximal to stem, and type C if distal to stem ( Figure 3 ).

Campbell et al.,^( 9 )^ proposed a classification based in 21 cases. The type 1 are tuberosity fractures, type 2, proximal metaphyseal fractures, type 3 humeral shaft fractures, and type 4, fracture distal to stem.

Worland et al.,^( 10 )^ classification describes fractures as following: type A fractures occur about the tuberosities, type B in to the level of stem, and B1 for spiral fractures with stable stem, B2 for oblique fractures about the tip of the stem (which is stable) and B3 for fractures about the stem with an unstable implant. Type C fractures occur distal to stem.

Our case would be classified as Wright et al., type C,^( 8 )^ Campbell et al., type 4^( 9 )^ and Worland et al., type C.^( 10 )^ This classifications do not guide treatment and nor establish prognosis of injuries. In addition, classifications can be useful in future studies with higher level of evidence to group similar fractures and compare methods of treatment.

The increase of RSA surgeries should be followed-up for its complications. Fracture reverse shoulder periarthroplasty fracture are injuries of difficult treatment both for technical reasons and comorbidities that patients often present.

## CONCLUSION

We describe a case that we decided for surgery of osteosynthesis with plate, screws and cerclage by lateral approach. Fracture healing was observed and reduction of active arch movement, as well as function. Surgical treatment of similar cases seemed to be the best option, although no studies exist including great samples.
